# Quantitative gastrointestinal function and corresponding symptom profiles in autonomic neuropathy

**DOI:** 10.3389/fneur.2022.1027348

**Published:** 2022-12-15

**Authors:** Jordan S. Langford, Eric Tokita, Cecilia Martindale, Leah Millsap, James Hemp, Laura A. Pace, Melissa M. Cortez

**Affiliations:** ^1^University of Utah School of Medicine, University of Utah, Salt Lake City, UT, United States; ^2^Department of Neurology, Imaging and Neurosciences Center, University of Utah, Salt Lake City, UT, United States; ^3^Metrodora Institute, West Valley City, UT, United States

**Keywords:** dysmotility, autonomic neuropathy, sudomotor dysfunction, COMPASS-31, gastrointestinal dysfunction

## Abstract

**Purpose:**

Peripheral neuropathies with autonomic nervous system involvement are a recognized cause of gastrointestinal dysmotility for a wide spectrum of diseases. Recent advances in wireless motility capsule testing allow improved sampling of regional and whole gut motility to aid in the diagnosis of gastrointestinal motility disorders and may provide additional insight into segment-specific enteric involvement of peripheral neuropathies affecting autonomic nervous system function.

**Methods:**

We utilized standardized autonomic nervous system (ANS) reflex assessment and wireless motility capsule testing to evaluate 20 individuals with idiopathic autonomic neuropathy and unexplained gastrointestinal symptoms. Additionally, we examined the relationship between quantifiable autonomic neuropathy and gastrointestinal dysmotility at specific neuroanatomical levels. Symptom profiles were evaluated using the 31-item Composite Autonomic Symptom Score questionnaire (COMPASS-31) and compared to wireless motility capsule data.

**Results:**

We found that transit times were predominately abnormal (delayed) in the foregut (10 of 20; 50%), while contractility abnormalities were far more prominent in the hindgut (17 of 20; 85%), and that motility and symptom patterns, as assessed by the COMPASS-31 GI domain items, generally corresponded. Finally, we also found that there was neuroanatomical overlap in the presence of autonomic reflex abnormalities and WMC-based transit and/or contractility abnormalities.

**Conclusions:**

We found that transit times were predominately abnormal in the foregut and midgut, while contractility abnormalities were far more prominent in the hindgut in individuals with idiopathic autonomic neuropathy. There was a high rate of agreement in segmental wireless motility capsule data with neuroanatomically corresponding standardized ANS function measures (e.g., cardiovagal, sudomotor, adrenergic). Expanded sudomotor testing, including additional neuroanatomical segments, could provide additional indirect assessment of visceral involvement in ANS dysfunction.

## Introduction

Gastrointestinal (GI) dysmotility is a recognized symptom of peripheral neuropathies with autonomic involvement, including in diabetes, systemic autoimmune disease, and hereditary conditions (1–4). Patients with idiopathic autonomic neuropathy (AN) also frequently present with symptoms affecting multiple organ systems, ranging from sensory, orthostatic, urinary, secretory, and gastrointestinal (GI) (1, 2, 5), though the functional patterns of GI dysmotility are poorly understood. GI motility is primarily controlled by the three branches of the autonomic nervous system *via* shared modulation from the parasympathetic and sympathetic nervous systems in addition to the intrinsic GI tract nervous system, known as the enteric nervous system (ENS) (2–6). While it is well established that ANS function plays an important role in GI motility and control of secretions (1–3, 6–8), there is marked diversity and complexity of symptoms among patients with autonomic disorders, often limiting the yield of conventional diagnostic clinical evaluations (5, 7). For example, symptoms of delayed or rapid gastric emptying can be ambiguous and difficult to distinguish clinically based on symptoms alone– therefore requiring objective testing in order to further delineate the underlying physiological pattern (5, 7, 9–12). The most common clinically utilized diagnostic assessments of GI motility allow only a limited, regional assessment of the proximal foregut or distal hindgut (e.g., esophageal manometry, gastric emptying, Sitz marker testing, and anorectal manometry), leaving large segments of the foregut and midgut uninterrogated (5). Recent advances in wireless motility capsule (WMC) testing now allow improved sampling of these previously uninterrogated regions, with the potential to improve diagnostic yield and (7, 9) provide valuable insight into segment-specific enteric involvement.

**Figure 1:**
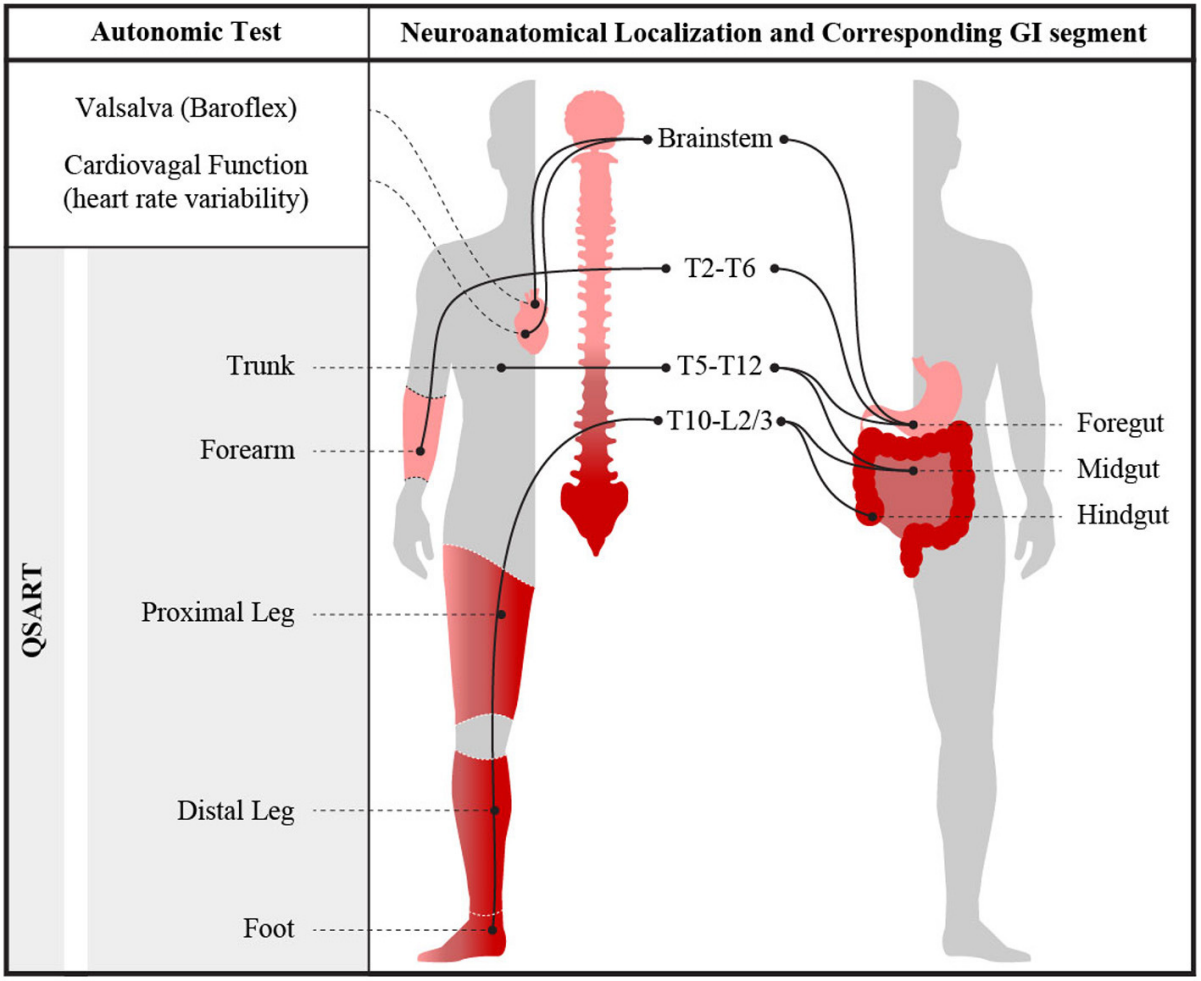
Visual representation of measured autonomic nervous system abnormalities obtained during ARS testing and their neuroanatomical segmental relationships. Illustration by Jeremy Theriot.

Clinically, AN is diagnosed based on a combination of presenting symptoms and expected features on standardized autonomic reflex screens (ARS). Such testing includes cardiovascular adrenergic and cardiovagal autonomic measures, as well as quantitative sudomotor axon reflex testing (QSART). Cardiovascular adrenergic and cardiovagal measures assess well-defined reflex arcs with primary neurons housed in the brainstem. QSART measures post-ganglionic (cholinergic) sympathetic function at pre-defined neuroanatomical levels (1, 2, 13–16), allowing for characterization of the distribution of peripheral sudomotor dysfunction (13, 15, 17, 18). Analogously, WMC can provide physiological measures of motility throughout each GI segment, further aiding in localization of autonomic neuropathic involvement in AN (3, 19). Thus, by comparing segmental WMC GI data with segmental QSART data, we aimed to examine the relationship between quantifiable AN and GI dysmotility at specific neuroanatomical levels (Figure 1). Additionally, we compared quantitative WMC data to symptomatic reports collected from patients using a standardized autonomic symptom assessment tool (COMPASS-31) to compare the relationship between physiological findings and reported symptomology (20). We hypothesize that GI dysmotility is both a localizable and quantifiable feature of AN.

## Materials and methods

### Group selection

We utilized an institutional database containing cases that presented to the University of Utah with undiagnosed GI and autonomic symptoms (2015–2019; *n* = 108); inclusion criteria for the database included complete ANS and WMC testing. Autonomic neuropathy diagnosis was defined based on abnormal findings on one or more of the following: abnormal cardiovagal function during deep breathing or Valsalva maneuver (VM) test and/or an abnormal QSART. Exclusion criteria for the AN group included: previous diagnosis or treatment of coronary artery disease, diabetes, and participants on any medications that were not withheld for at least 48 h and/or could interfere with autonomic testing results. A total of 24 subjects met inclusion criteria for AN; two were excluded due to missing autonomic testing data, and two were removed due to possible medications effects. Thus, a total of 20 subjects met AN criterion and were included in this analysis.

### Motility testing

Wireless motility capsule (WMC) testing was administered using the Smartpill^TM^ Motility test, based on published protocols (Medtronic, Minneapolis, MN) (10, 21). WMC results were analyzed for transit times and contractility. WMC based transit times were compared to published normative ranges of gastric emptying time, small bowel transit time (SBTT), colonic transit time (CTT) and whole gut transit times (WGTT) (10–12, 21) for each subject. Given the heterogeneity of methods used to determine normative ranges, cut-off parameters for abnormal vs. normal contractility patterns segmental transit times were based upon values falling outside of published metrics, inclusive of both excessively rapid and/or delayed transit times, rather than on percentiles. Cut-off parameters for abnormal vs. normal were based on published values and were considered abnormal if they fell below the 5th or above 95th percentiles respectively (22). Reference ranges used to determine abnormal transit times on WMC testing is summarized in Table 1.

**Table 1:** Reference ranges for WMC testing (10–12, 21).

**WMC transit time parameters**	
**Gastrointestinal segment**	**Normal transit time (h)**
Gastric emptying time (GET)	2.5–4.5
Small bowel transit time (SBTT)	2.5–6.0
Colonic transit time (CTT)	10–59
Whole gut transit time (WGTT)	15–73

### Autonomic testing

#### Cardiovagal and cardiovascular testing

The severity and distribution of autonomic dysfunction was quantified using the composite autonomic severity score (CASS) score, where 0 indicates minimal to no dysfunction and 10 indicating maximum autonomic impairment. The CASS score is further divided into three sub-scores: sudomotor, cardiovagal, and adrenergic; normalized for confounding effects of age and gender (23).

Cardiovagal and cardiovascular adrenergic autonomic function were assessed *via* previously published methods (16, 24). In brief, cardiovagal function was investigated using heart rate changes with deep breathing (HRDB) and Valsalva ratio (VR). HRDB was calculated after eight cycles of breathing at a rate of 6 breaths per minute. Subjects were provided a cue of an oscillating arrow to aid in achieving inspiratory and expiratory cycles of 5 s; subjects completed two trials, separated by 2 min of supine rest. HRDB was then calculated, based on the average of the highest five consecutive cycles. An abnormal result was based on published normative age-dependent ranges of beats per minute (BPM) during HRDB (25). During Valsalva Maneuver (VM) subjects were asked to inhale deeply and then exhale into a bugle that contains an air leak (WR Medical Electronics Co., Maplewood, MN) with the goal of maintaining an expiratory pressure of 40 mmHg for a minimum of 15 s. VR was calculated by dividing the maximum heart rate by the minimum heart rate that occurred within the first 30 s of releasing the VM. An abnormal VR was based off published age-dependent normative ranges for age and sex (25). Abnormal cardiovagal function was thus associated with a CASS vagal subscore of 1 or greater.

Cardiovascular adrenergic autonomic function was examined by blood pressure (BP) and Heart Rate (HR) changes to head up tilt (HUT) and Valsalva maneuver (VM). For HUT testing, subjects were placed in a supine position for 20 min before obtaining a 5 min baseline recording of HR and BP. Subjects were then tilted to 70 degrees above horizontal for 10 min before returning to a supine position for an additional 5 min. Subjects' HR and BP were measured using continuous beat-to-beat BP device (CNAP^®^, CNSystems Medizintechnik GmbH, Graz, Austria). Postural HR change was calculated by taking the difference between the average heart rate in the supine position and a 30 s average of the maximal HR in the upright position. Abnormal BP responses to Valsalva were based on excessive early phase II decrease and/or abnormal phase IV according to published CASS adrenergic scoring guidelines (23). An abnormal BP change to tilt was defined as a systolic decrease of >20 mmHG or decrease of >10 mmHG at 3 min (15). Thus, abnormal cardiovascular adrenergic function was associated with, and confirmed by, a CASS score of 1 or greater in the adrenergic subscore of the CASS. All data was collected using WR TestWorks™ software (WR Medical Electronics Co., Stillwater, MN).

#### QSART testing

QSART was conducted using previously published methods (26). At a resting state, patients were assessed for sweat function response in the medial forearm (dermatome level C6–T2), proximal leg (dermatome level L1–L3), distal leg (dermatome level L3–L5), and lateral foot (dermatome level S1). At each location, the skin was exfoliated with sandpaper, then cleaned using alcohol, acetone, and a washcloth before attaching the sudorometer-containing electrodes (WR Medical Electronics Co., Maplewood, MN). An electrical current of 2 mA was applied for 5 min, during which acetylcholine was iontophoresed into the skin. Onset and output of sweat was measured for 5 additional minutes, for a total of a 10 min recording. TestWorks™ version 3.4 was used to analyze the sudomotor data. Test results were interpreted by a specialty-trained physician [MC] and compared to published age and sex-based normative data, where a 5th percentile cut-off was used to identify abnormal results for each site (27). Abnormal sudomotor function was associated with a CASS sudomotor subscore of 1 or greater.

### Symptomatic evaluation *via* COMPASS-31

All but one subject (*n* = 19) completed a validated patient report symptom assessment, the 31-item Composite Autonomic Symptom Score (COMPASS-31) questionnaire (20). COMPASS-31 assesses self-reported symptoms across 6 domains: orthostatic intolerance, vasomotor, secretomotor, gastrointestinal, bladder, and pupillomotor. Each domain is scored based on severity and frequency. A total summary score is collected based on the weighted score from each domain with a higher score suggestive of increased symptomatic burden. Scores from our subject population were then compared with previously published healthy control ranges (28). For symptom correlation with WMC findings, subjects were assigned designation of experiencing significant diarrhea or constipation based off their responses to the severity/frequency of their symptoms. Those who self-reported “frequent” or “constant” symptoms in in items #16–18 and #20–22 respectively were considered clinically significant, and used to evaluate correspondence with WMC findings (Results section Autonomic symptoms).

### Neuroanatomical organization

To evaluate for neuroanatomical parsimony between the autonomic reflex test results and WMC abnormalities, HRDB, VR, and Valsalva-based BP abnormalities (CASS-vagal and CASS-adrenergic), as well as QSART abnormalities in the forearm, were taken to correspond with foregut, whereas QSART testing in the proximal leg, distal leg, and foot were expected to correspond with the level of the hindgut (6, 19, 29–32). Visual representation of the neuroanatomical relationship between gastric and autonomic sudomotor innervation is shown in Figure 1. HRDB, Valsalva, and QSART data from each individual were compared to their WMC transit times and contractility data to identify areas of autonomic reflex and GI dysfunction with overlapping neuroanatomical levels. Gastric transit times (GET) and contractility were used to measure foregut dysfunction; while SBTT and CTT and small bowel/colonic contractility were used to measure midgut and hindgut dysfunction.

## Results

The average age of our test group was 35 years (range 17–62, SD 13.3) with 3 males and 17 females (15 and 85% respectively).

### Autonomic testing

Seventeen of 20 (85%) individuals had abnormal QSART testing results. Four of 20 (20%) had cardiovagal impairment determined by abnormal HRDB response or VR during autonomic testing. Based on abnormal Valsalva and/or BP response during head up tilt test, 13 (65%) had cardiovascular adrenergic impairment. Autonomic testing results and characteristics are summarized in Table 2.

**Table 2:** Summary of ARS and WMC testing and symptomatic profiles.

**Autonomic reflex study**					**Wireless mobile capsule**					**Symptomatic profile**	
**Subject**	**CASS_Total_**	**CASS_S_**	**CASSv**	**CASSa**	**GET**	**SBTT**	**CTT**	**WGTT**	**Contractility**	**COMPASS−31_Composite_**	**Diarrhea/constipation**
1	2	***1** ^***F***^*	0	**1**	***24.1** ^***D***^*	**8.9** ^D^	***66.6** ^***D***^*	***99.5** ^***D***^*	**Incr CC**	49	**Frequent**/No
2	1	0	***1***	0	***2.3** ^***R***^*	4.3	17.5	24.0	***Incr CC***	*79*	**Frequent/constant**
3	3	***2** ^***FA, F***^*	0	***1***	***0.4** ^***R***^*	6.0	24.8	31.2	***Incr CC***	*24*	**Frequent/constant**
4	3	***2** ^***FA, PL***^*	0	***1***	***1.5** ^***R***^*	***9.3** ^***D***^*	20.2	31.0	***Incr CC***	*45*	No/**frequent**
5	4	***3** ^***FA, PL***^*	0	***1***	***1.8** ^***R***^*	3.2	***8.1** ^***R***^*	***13.2** ^***R***^*	***Incr CC***	*41*	**Constant**/no
6	4	***3** ^***FA, PL, DL, F***^*	0	***1***	2.9	4.8	29.6	37.3	***Incr CCs***	*57*	No/**constant**
7	***1***	0	***1***	0	***7.1** ^***D***^*	***12.5** ^***D***^*	28.7	48.3	***Incr CC***	*65*	**Frequent/frequent**
8	1	***1** ^***F***^*	0	0	***22.3** ^***D***^*	***6.5** ^***D***^*	***92.2** ^***D***^*	***121.0** ^***D***^*	***Incr CC***	*55*	No/**constant**
9	2	***2** ^***F***^*	0	0	3.3	2.8	23.4	29.5	***Inc CC***	*90*	**Constant**/no
10	4	***2** ^***PL, DL***^*	0	***2***	***2.0** ^***R***^*	4.2	18.2	24.9	***Incr CC***	*39*	No/**frequent**
11	2	***1** ^***PL***^*	0	***1***	3.6	***7.3** ^***D***^*	19.9	30.9	Normal	*47*	No/no
12	6	***2** ^***F***^*	***1***	***3***	***5.6** ^***D***^*	3.6	27.6	36.8	***Incr CC***	*51*	**Constant/frequent**
13	3	***3** ^***PL, DL***^*	0	0	2.8	3.0	16.0	21.8	***Incr CC***	*71*	**Frequent/frequent**
14	***2***	0	***1***	***1***	***18.4** ^***D***^*	***6.8** ^***D***^*	***60.0** ^***D***^*	***74.8** ^***D***^*	***Decr CC***	*26*	No/no
15	2	***2** ^***PL, DL***^*	0	0	***5.8** ^***D***^*	4.3	44.5	54.6	***Incr SC***	*57*	No/**constant**
16	2	***1** ^***F***^*	0	***1***	***4.6** ^***D***^*	4.1	17.6	26.2	Normal	*62*	**Constant/constant**
17	3	***2** ^***F***^*	0	***1***	***4.6** ^***D***^*	5.4	18.1	28.1	***Incr CC***	*53*	**Frequent**/no
18	2	***1** ^***F***^*	0	***1***	***5.6** ^***D***^*	5.5	7.7	18.8	***Incr CC***	*61*	**Frequent**/no
19	2	***2** ^***FA, PL, DL***^*	0	0	***1.40** ^***R***^*	4.8	40.8	47.2	***Incr CC***	*81*	**Constant**/no
20	4	***2** ^***F, PL, DL***^*	***1***	***1***	***5.6** ^***D***^*	2.9	44.4	53.0	***Incr CC***	NA	NA

CC, colonic contractions; D, delayed; Decr, decreased; DL, distal leg; F, foot; FA, forearm; Incr, increased; PL, proximal leg; R, rapid; SC, stomach contractions. Bolded for assignation of abnormal values.

### WMC results

Summary of transit time and contractility results can be seen in Table 2. Of the 20 study participants with AN and undiagnosed GI symptoms, 17 (85%) had objectively quantifiable abnormal segmental transit times, with a mean of 1.8 (range 0–4) affected segments. Delayed transit times accounted for the majority of abnormal segments with 12 of 20 (60%) individuals having at least one delayed segment on WMC testing; while 6 of 20 (30%) exhibited abnormally rapid transit times in one or more segments. Delayed gastric emptying was the most common segmental finding, which was abnormal in 10 of 20 (50%), followed by delayed SBTT and rapid GET both at 6 (30%), delayed CTT and delayed WGTT at 3 (15%).

Of the 20 study participants, 18 of 20 (90%) had objectively quantifiable abnormal contractility recordings, with a mean of 1 (range 0–1) affected segment. Of these 17of 20 (85%) individuals had increased contractility in the large bowel (hindgut) and 1 (5%) had increased stomach contractions (foregut), whereas only 1/20 (5%) had decreased contractions, which were in the small bowel (midgut).

### Autonomic symptoms

As seen in Table 2, our AN group had a median summary COMPASS-31 score of 55.4 (range 24–90), with a median score of 16.3 for the GI domain. In our cohort, 11 complained of frequent or constant constipation, 12 frequent or constant diarrhea, and 6 reported both.

COMPASS-31 item responses were used to investigate the relationship between patient-reported symptomatic disease and objective abnormal transit times and contractility. We found that 6/12 (50%) individuals that had one or more quantifiable segmental delay also reported symptoms of constipation (items #20–22 within the questionnaire). Additionally, 4/6 (67%) individuals that had at least one quantifiable rapid segment transit time, also reported symptoms of diarrhea (items 16–18 within the questionnaire). Interestingly, 11 of 15 (73%) individuals with increased contractility in the colon on WMC testing reported symptoms of diarrhea. However, as might be clinically expected, the 1 individual who had decreased colonic contractility did not report symptomatic constipation.

### Autonomic reflex testing and segmental WMC abnormalities

Fifteen of 17 (88%) of those with an abnormal QSART site demonstrated abnormal transit times or contractility in the respective GI segment. Of the 17 subjects who had abnormal QSART data in the proximal leg, distal leg or foot corresponding to neuroanatomical level of the midgut/hindgut, 15/17 (88%) also had abnormal CTT or colonic contractility. Of the 6 individuals with abnormal forearm QSART, 5 of 6 (83%) had abnormal GET. 4/4 (100%) cardiovagal and 11/13 (85%) cardiovascular adrenergic impairment (CASS > 1 for vagal and/or adrenergic domains) had corresponding abnormal GET. In total, subjects with at least one abnormality in forearm QSART, CASS_V_, and CASS_A_ 14/16 (88%) had abnormal autonomic reflex abnormalities corresponding to neuroanatomical level of the foregut.

## Discussion

Our study utilizes physiological data obtained from WMC and standard AN testing to identify gastrointestinal dysfunction in patients with AN. We found that transit times were predominately abnormal (delayed) in the foregut (10 of 20; 50%), while contractility abnormalities were far more prominent in the hindgut (17 of 20; 85%), and that motility and symptom patterns, as assessed by the COMPASS-31 GI domain items, generally corresponded. Finally, we also found that there was neuroanatomical overlap in the presence of autonomic reflex abnormalities and WMC-based transit and/or contractility abnormalities.

### GI dysfunction in autonomic disease

The clinical manifestations of autonomic impairment and related GI dysfunction have been well documented in a variety of diseases where autonomic neuropathy is recognized, including diabetes mellitus and Parkinson's disease (5, 8, 33–35). Our study aimed to further characterize this relationship in subjects with idiopathic AN and evaluate associations between quantitative GI motility and autonomic assessments. Our data further confirms that GI dysfunction is a common and quantifiable aspect of AN. The comprehensive data obtained from WMC testing allows us to examine each segment of the GI tract to quantify changes in motility and contractility. In concordance with other studies examining motility patterns in autonomic disease states (33, 34, 36), abnormal gastric emptying was the most common finding of transit abnormality among our AN cohort (78% with abnormal gastric emptying time). Additionally, abnormal contractility (generally increased) in the hindgut (74%), was common in our study. These findings suggest that symptomatic GI dysfunction due to AN disease affects more than just the foregut and therefore requires a pan-enteric diagnostic approach.

The relatively high summary COMPASS-31 score and median GI domain score for our AN group is comparable to previously published COMPASS median scores in AN patients, and clearly greater than a median total score of 12.6 in healthy controls (26). Interestingly, the median GI domain score in our cohort was somewhat greater than previously published AN GI domain scores (median of 12.0) (28), though this is not entirely unexpected, given an inclusion criterion for our database was of otherwise unexplained GI symptoms. Future studies examining GI motility in an unselected sample of AN patients would help clarify whether the patterns seen in our cohort are common, or whether our sample simply represents a subset of patients with more prominent GI involvement than expected.

Clinical equipoise remains as to the practical utility of objective characterization of GI dysfunction in autonomic disease states (5). Some studies suggest that symptoms and objective GI testing data are not consistently related, nor do they clearly guide management (5, 35, 37). However, our results show that domain-specific symptom reports, *via* the COMPASS-31, indeed associate with abnormal patterns on WMC testing, and that the WMC based abnormalities frequently co-present with quantifiable and neuroanatomically corresponding changes in autonomic reflex measures. WMC provides multiple modalities (transit and contractility reported here; as well as other secretory metrics not evaluated in this analysis) to quantitative study GI dysfunction and does so in a minimally invasive approach and without radiation exposure, providing insight into a physiological basis for symptoms and objective confirmation of underlying visceral autonomic involvement in disorders of autonomic function. Additionally, studies have shown that medical decision-making based on WMC data have improved treatment outcomes (9, 36). Thus, we conclude that a pan-colonic enteric diagnostic approach to patients with AN who present with otherwise unexplained symptoms of GI dysfunction may be warranted.

### Segment of motility dysfunction corresponds with autonomic testing

Despite the heterogenous, multisystem nature of AN symptoms, the organization of the ANS provides a framework to facilitate localization of end organ (visceral) dysfunction based on its known neuroanatomical structure (4, 38, 39). For example, damage to autonomic pathways within the spinal cord, such as in multiple sclerosis or spinal cord trauma, has shown corresponding segmental GI dysmotility at the level of the lesion (40). Unfortunately, direct, objective diagnostic tests for enteric neuropathy are not routinely available clinically, and prior reports note a discordance between GI symptoms and objective motility dysfunction (5). One potential opportunity is to utilize existing, standardized autonomic testing as a surrogate to assess the functional integrity of neural pathways that also influence gut function (5).

Parasympathetic preganglionic neurons reside primarily in the brainstem and the distal sacral spinal cord (S2–S4) (3, 4, 16, 41). In particular, the vagal nerve (originating in the medulla) provides parasympathetic innervation to the foregut, while the distal sacral spinal cord provides parasympathetic innervation to the hindgut (3, 16, 19, 41). The preganglionic cell bodies of the sympathetic portion of the ANS reside in the thoracolumbar spinal cord between the T1/T2 spinal segments and L2 spinal segment (3, 4, 19). The sympathetic tracts within the spinal cord can be further anatomically categorized into the following innervation pattern: spinal cord levels T6–T9 provide sympathetic innervation to the foregut, T10–T12 innervate midgut and L2–L5 primarily innervate hindgut (3, 4, 6, 19, 32, 42) (Figure 1). Current clinically available standardized autonomic testing is based on well-described reflex pathways involving and overlapping with this neuroanatomical organization; analogously, regionally directed gastrointestinal motility testing could be considered an extension of ANS testing, with additional localizing ability. Here, sudomotor dermatomes evaluated by the current QSART protocol include: T2–T6 in upper limb and T10–L2/L3 in the lower limb (18). QSART testing in forearm could be expected to correspond to the foregut while the proximal leg, distal leg, and foot could be expected to correspond with the level of the midgut and hindgut (6, 18, 19, 31, 32, 42, 43). Abnormal cardiovagal and cardiac adrenergic function were taken to correspond with brainstem-mediated innervation of the foregut (4).

Previous authors have suggested using cardiac autonomic neuropathy as a marker for enteric nervous system (ENS) involvement; however, this relationship is not well understood (5, 8). In our study, 78% of individuals with AN presenting with unexplained GI symptoms had abnormal transit times in the foregut, with delayed gastric emptying being the most common finding. It is known that the vagal nerve and parasympathetic nervous system have an excitatory effect on gastric motility (19), and thus it is unsurprising that subjects with AN-mediated vagal impairment have a parallel decrease in gastric motility. In fact, our study data support a direct relationship between vagal dysfunction, as shown on AN testing, and corresponding foregut dysfunction. Of note, though foregut transit times were abnormal, foregut contractility data was overwhelmingly unremarkable. This could suggest a possible compensatory mechanism in the foregut that results in abnormal transit times with preserved number of contractions and gastric muscular function. The delayed transit times in our cohort could be explained by a lack of regular input from the parasympathetic nervous system. This lack of neuromodulation could explain why transit times are delayed, but when stimulated, the foregut is able to contract appropriately.

Additionally, individuals with post-ganglionic sympathetic autonomic dysfunction, as measured by QSART, also exhibited neuroanatomically congruent abnormalities on WMC assessment (79% of those with an abnormal QSART site demonstrated abnormal transit times or contractility in the respective GI segment). The dermatomal nature of QSART allows for measurement of sudomotor function corresponding with specific sympathetic spinal cord levels (26). An important distinction in this data is that abnormal transit times in the hindgut were only present in 6 of the subjects, while abnormally increased hindgut contractility was present in all subjects with abnormal lower limb QSART, in a dermatomal pattern that refers to spinal levels contributing to hindgut innervation. This is a direct contrast to the parasympathetic and foregut transit-contractility data discussed above. It is widely accepted that sympathetic activation inhibits gastric motility and contractility (19). The increased colonic contractility could be explained by the loss of inhibitory sympathetic input to that region, resulting in increased colonic motility and contractility. This would support the relationship between the loss of sympathetic input seen on QSART and the abnormal WMC data in the hindgut. Conversely, the increased contractility seen in the hindgut could be a compensatory mechanism for overall decreased GI function. These findings suggest possible neuroanatomic agreement across visceral (GI) and peripheral (post-ganglionic sudomotor) manifestations of AN. However, this use of cardiovagal and sympathetic sudomotor assessment only serves as a proxy for autonomically mediated GI function. Given that WMC testing availability remains scarce, and standardized manometry and gastric emptying studies may be segmentally insufficient for full symptomatic-functional correlation, additional investigative strategies are needed to better understand the clinical implications of the above findings. Future studies involving full thickness gastrointestinal tract biopsies subjected to ENS mapping *via* histopathology may prove very useful in enhancing the diagnostic yield for enteric neuropathies.

### Limitations

Current testing protocols do not include autonomic reflex testing in all relevant neuroanatomical locations, which has limited our neuroanatomical comparison. In particular, segments corresponding with midgut innervation are underrepresented. This shortcoming limited our ability to analyze agreement between autonomic reflex and GI dysfunction at the midgut, although this could be further explored using full body sudomotor testing, such as thermoregulatory sweat testing (15, 44).

Another potential limitation may arise from selection bias within of our cohort. All individuals included in our cohort presented to a quaternary medical center with both GI and autonomic symptoms and were found to have autonomic neuropathy. We did not include those with a previously known AN diagnosis, such as diabetic AN, nor those with AN and absent clinically significant GI symptoms. Thus, the neuroanatomical correspondence of GI function and autonomic testing in all-comer AN and/or additional specific autonomic disease states should be further evaluated. Additionally, our subject population is extremely symptomatic and as a result, the symptomatic correlation between objective and subjective findings could be skewed and not accurately represent the broader population.

## Conclusions

In summary, the combination of autonomic reflex and WMC testing provides a (near-) comprehensive, segmental assessment of GI motility in patients with AN and unexplained GI symptoms. We found that transit times were predominately abnormal in the foregut, while contractility abnormalities were far more prominent in the hindgut. Notably, there was a strikingly high rate of correspondence in segmental transit and/or contractility measures with neuroanatomically corresponding standardized autonomic function measures (e.g., cardiovagal, sudomotor, adrenergic). Expanding sudomotor testing to include dermatomes that correlate to foregut and midgut could provide further information of visceral involvement and assist in further validation of sudomotor dysfunction as a potential marker of ENS disease.

## Data availability statement

The raw data supporting the conclusions of this article will be made available by the authors, without undue reservation.

## Ethics statement

The studies were a secondary analysis of an existing database and the study procedures were reviewed and approved by the University of Utah institutional review board. The requirement for written consent was waived.

## Author contributions

The authors confirm the contributions to this paper as follows: study conception and design: JL, MC, and LP. Analysis and interpretation of results: JL, ET, JH, LP, and MC. Draft manuscript preparation: JL, ET, LM, CM, JH, LP, and MC. All authors reviewed the results and approved the final version of the manuscript.

## References

[1] Freeman R. Autonomic peripheral neuropathy. Continuum. (2020) 26:58–71. 10.1212/CON.0000000000000825 31996622

[2] Novak P. Autonomic disorders. Am J Med. (2019) 132:420–36. 10.1016/j.amjmed.2018.09.027 30308186

[3] Benarroch EE. Enteric nervous system: functional organization and neurologic implications. Neurology. (2007) 69:1953–7. 10.1212/01.wnl.0000281999.56102.b5 17998487

[4] Benarroch EE. Physiology and pathophysiology of the autonomic nervous system. Continuum. (2020) 26:12–24. 10.1212/CON.0000000000000817 31996619

[5] Kornum DS Terkelsen AJ Bertoli D Klinge MW Hoyer KL Kufaishi HH. Assessment of gastrointestinal autonomic dysfunction: present and future perspectives. J Clin Med. (2021) 10:71392. 10.3390/jcm10071392 33807256 PMC8037288

[6] Furness JB Callaghan BP Rivera LR Cho HJ. The enteric nervous system and gastrointestinal innervation: Integrated local and central control. Adv Exp Med Biol. (2014) 817:39–71. 10.1007/978-1-4939-0897-4_3 24997029

[7] Grover M Farrugia G Stanghellini V. Gastroparesis: a turning point in understanding and treatment. Gut. (2019) 68:2238–50. 10.1136/gutjnl-2019-318712 31563877 PMC6874806

[8] Nguyen L Wilson LA Miriel L Pasricha PJ Kuo B Hasler WL. Autonomic function in gastroparesis and chronic unexplained nausea and vomiting: relationship with etiology, gastric emptying, and symptom severity. Neurogastroenterol Motil. (2020) 32:e13810. 10.1111/nmo.13810 32061038 PMC7377964

[9] Hasler WL Rao SSC McCallum RW Krause RA Nguyen LA Schulman MI. Influence of gastric emptying and gut transit testing on clinical management decisions in suspected gastroparesis. Clin Transl Gastroenterol. (2019) 10:e00084. 10.14309/ctg.0000000000000084 31663906 PMC6919448

[10] Kuo B McCallum RW Koch KL Strin MD Wo JM Chey WD. Comparison of gastric emptying of a nondigestible capsule to a radio-labelled meal in healthy and gastroparetic subjects. Aliment Pharmacol Therapeut. (2008) 27:186–96. 10.1111/j.1365-2036.2007.03564.x 17973643

[11] Rao SSC Kuo B McCallum RW DiBoise JK Hasler WL Koch KL. Investigation of colonic and whole-gut transit with wireless motility capsule and radiopaque markers in constipation. Clin Gastroenterol Hepatol. (2009) 7:537–44. 10.1016/j.cgh.2009.01.017 19418602

[12] Tougas G Eaker EY Abell TL Abrahamsson H Boivin M Chen J. Assessment of gastric emptying using a low fat meal: establishment of international control values. Am J Gastroenterol. (2000) 95:1456–62. 10.1111/j.1572-0241.2000.02076.x 10894578

[13] Riedel A Braune S Kerum G Schulte-Mönting J Lücking CH. Quantitative sudomotor axon reflex test (QSART): a new approach for testing distal sites. Muscle Nerve. (1999) 22:1257–64. Available online at: 10.1002/(SICI)1097-4598(199909)22:9<1257::AID-MUS14>3.0.CO;2-J 10454723

[14] Hu Y Converse C Lyons MC Hsu WH. Neural control of sweat secretion: a review. Br J Dermatol. (2018) 178:1246–56. 10.1111/bjd.15808 28714085

[15] Freeman R Chapleau MW. Testing the autonomic nervous system. Handb Clin Neurol. (2013) 115:115–36. 10.1016/B978-0-444-52902-2.00007-2 23931777

[16] Low PA. Testing the autonomic nervous system. Semin Neurol. (2003) 23:407–21. 10.1055/s-2004-817725 15088262

[17] Ziemssen T Siepmann T. The investigation of the cardiovascular and sudomotor autonomic nervous system: a review. Front Neurol. (2019) 10:53. 10.3389/fneur.2019.00053 30809183 PMC6380109

[18] Mathias CJ Bannister R. Autonomic Failure: A Textbook of Clinical Disorders of the Autonomic Nervous System, 5th ed. Oxford: Oxford Academic (2013). 10.1093/med/9780198566342.001.0001

[19] Browning KN Travagli RA. Central nervous system control of gastrointestinal motility and secretion and modulation of gastrointestinal functions. Compreh Physiol. (2014) 4:1339–68. 10.1002/cphy.c130055 25428846 PMC4858318

[20] Sletten DM Suarez GA Low PA Mandrekar J Singer W COMPASS. 31: a refined and abbreviated composite autonomic symptom score. Mayo Clin Proc. (2012) 87:1196–201. 10.1016/j.mayocp.2012.10.013 23218087 PMC3541923

[21] Rao SSC Camilleri M Hasler WL Maurer AH Parkman HP Saad R. Evaluation of gastrointestinal transit in clinical practice: position paper of the American and European neurogastroenterology and motility societies. Neurogastroenterol Motil. (2011) 23:8–23. 10.1111/j.1365-2982.2010.01612.x 21138500

[22] Farmer AD Wegeberg AML Brock B Maurer AH Parkman HP Saad R. Regional gastrointestinal contractility parameters using the wireless motility capsule: inter-observer reproducibility and influence of age, gender and study country. Aliment Pharmacol Therapeut. (2018) 47:391–400. 10.1111/apt.14438 29210098

[23] Low PA. Composite autonomic scoring scale for laboratory quantification of generalized autonomic failure. Mayo Clin Proc. (1993) 68:748–52. 10.1016/S0025-6196(12)60631-4 8392653

[24] Cohen JA. Clinical autonomic disorders, third edition. Neurology. (2010) 74:617. 10.1212/WNL.0b013e3181cff7c3

[25] Low P. Laboratory evaluation of autonomic function. In: Clinical Autonomic Disorders. 2nd, ed. Philadelphia, PA: Lippincott – Raven (1997). p. 182–5.

[26] Ravits JM. AAEM minimonograph #48: autonomic nervous system testing. Muscle Nerve. (1997) 20:919–37. Available online at: 10.1002/(SICI)1097-4598(199708)20:8<919::AID-MUS1>3.0.CO;2-9 9236782

[27] Sletten D Grandinetti A Weigand S Gehrking T Gehrking J Low P Singer W. Normative values for sudomotor axon reflex testing using QSWEAT^TM^ (P1.282). Wolfgang Singer. Neurology. (2015) 84(14 Supplement):P1.282. Available online at: https://n.neurology.org/content/84/14_Supplement/P1.282

[28] Rea NA Campbell CL Cortez MM. Quantitative assessment of autonomic symptom burden in Postural tachycardia syndrome (POTS). J Neurol Sci. (2017) 377:35–41. 10.1016/j.jns.2017.03.032 28477704

[29] Cheshire WP Freeman R Gibbons CH Cortelli P Wenning GK Hilz MJ. Electrodiagnostic assessment of the autonomic nervous system: a consensus statement endorsed by the American Autonomic Society, American Academy of neurology, and the international federation of clinical neurophysiology. Clin Neurophysiol. (2021) 132:666–82. 10.1016/j.clinph.2021.02.006 33419664

[30] Freeman R Wieling W Axelrod FB Benditt DG Benarroch E Biaggioni I. Consensus statement on the definition of orthostatic hypotension, neurally mediated syncope and the postural tachycardia syndrome. Autonom Neurosci Basic Clin. (2011) 161:46–8. 10.1016/j.autneu.2011.02.004 21393070

[31] Longo WE Ballantyne GH Modlin IM. The colon, anorectum, and spinal cord patient. A review of the functional alterations of the denervated hindgut. Dis Colon Rectum. (1989) 32:261–7. 10.1007/BF02554543 2646085

[32] Jänig W McLachlan EM. Organization of lumbar spinal outflow to distal colon and pelvic organs. Physiol Rev. (1987) 67:1332–404. 10.1152/physrev.1987.67.4.1332 2891149

[33] Zhou W Zikos TA Clarke JO Nguyen LA Triadafilopoulos G Neshatian L. Regional gastrointestinal transit and contractility patterns vary in postural orthostatic tachycardia syndrome (POTS). Dig Dis Sci. (2021) 66:4406–13. 10.1007/s10620-020-06808-z 33428036

[34] Lee AA Rao S Nguyen LA Moshiree B Sarosiek I Schulman MI. Validation of diagnostic and performance characteristics of the wireless motility capsule in patients with suspected gastroparesis. Clin Gastroenterol Hepatol. (2019) 17:1770–9.e2. 10.1016/j.cgh.2018.11.063 30557741 PMC7442471

[35] Knudsen K Fedorova TD Bekker AC Iversen P Ostergaard K Krogh K. Objective colonic dysfunction is far more prevalent than subjective constipation in Parkinson's disease: a colon transit and volume study. J Parkinson's Dis. (2017) 7:359–67. 10.3233/JPD-161050 28157109

[36] Rouphael C Arora Z Thota PN Lopez R Santisi J Funk C. Role of wireless motility capsule in the assessment and management of gastrointestinal dysmotility in patients with diabetes mellitus. Neurogastroenterol Motility. (2017) 29:13087. 10.1111/nmo.13087 28444862

[37] Bharucha AE Camilleri M Forstrom LA Zinsmeister AR. Relationship between clinical features and gastric emptying disturbances in diabetes mellitus. Clin Endocrinol. (2009) 70:415–20. 10.1111/j.1365-2265.2008.03351.x 18727706 PMC3899345

[38] Prévinaire JG Mathias CJ El Masri W Soler JM Leclercq V Denys P. The isolated sympathetic spinal cord: cardiovascular and sudomotor assessment in spinal cord injury patients: a literature survey. Ann Phys Rehabil Med. (2010) 53:520–32. 10.1016/j.rehab.2010.06.006 20797928

[39] Reitz A Schmid DM Curt A Knapp PA Schurch B. Sympathetic sudomotor skin activity in human after complete spinal cord injury. Auton Neurosci. (2002) 102:78–84. 10.1016/S1566-0702(02)00207-2 12492139

[40] Preziosi G Raptis DA Raeburn A Thiruppathy K Panicker J Emmanuel A. Gut dysfunction in patients with multiple sclerosis and the role of spinal cord involvement in the disease. Eur J Gastroenterol Hepatol. (2013) 25:1044–50. 10.1097/MEG.0b013e328361eaf8 23669324 PMC4206376

[41] Taché Y Yang H Kaneko H. Caudal raphe-dorsal vagal complex peptidergic projections: role in gastric vagal control. Peptides. (1995) 16:431–5. 10.1016/0196-9781(94)00212-O 7544464

[42] Loukas M Klaassen Z Merbs W Tubbs RS Gielecki J Zurada A. review of the thoracic splanchnic nerves and celiac ganglia. Clin Anatomy. (2010) 23:512–22. 10.1002/ca.20964 20235178

[43] Jänig W. Integration of gut function by sympathetic reflexes. Baillieres Clin Gastroenterol. (1988) 2:45–62. 10.1016/0950-3528(88)90020-6 2838110

[44] Cheshire WP. Sudomotor dysfunction. Semin Neurol. (2020) 40:560–8. 10.1055/s-0040-1713847 32906168

